# Supplementary material for: Geometric constraint-triggered collagen expression mediates bacterial-host adhesion
Source: Nat Commun. 2023 Dec 9;14:8165. doi: 10.1038/s41467-023-43827-6 (PMC10710423; doi:10.1038/s41467-023-43827-6)
Supplement: Supplementary file 1 — Supplementary Information [file 41467_2023_43827_MOESM1_ESM.pdf]

Supplementary Information for

Geometric constraint-triggered collagen expression mediates bacterial-host adhesion

Supplementary Information includes:

Supplementary Figures 1 to 11

Supplementary Fig. 1 Dependence of spatially heterogeneous bacteria-cell adhesion on geometric constraints.

Supplementary Fig. 2 Spatiotemporal distribution of bacterial adhesion to geometrically constrained cell monolayers.

Supplementary Fig. 3 Bacteria-cell heterogeneous adhesion phenomenon independence of cell types and adhesive substance coated on PAAm substrates.

Supplementary Fig. 4 Dependence of adhesion forces between single bacterium and micropatterned host cell monolayers on geometric constrains.

Supplementary Fig. 5 Single-cell RNA sequencing analysis.

Supplementary Fig. 6 Spatial distribution of collagen I and collagen II on the micropatterned host cell monolayers.

Supplementary Fig. 7 Edge effects of collagen IV on the micropatterned host cell monolayers.

Supplementary Fig. 8 Spatially heterogeneous expression of vinculin and collagen IV in the micropatterned cell monolayers and spatial distribution of cellular traction forces.

Supplementary Fig. 9 Monte Carlo simulations of bacteria-cell adhesion under geometric constrains.

Supplementary Fig. 10 Collagen IV inhibitors enhanced the effect of antibiotics and promoted wound healing.

Supplementary Fig. 11 Dependence of invasion of *S. aureus* to IEC-6 cell monolayers on substrate rigidities.

Supplementary Tables 1 to 3

Supplementary Table 1. Statistical results of single cell areas at the central and peripheral regions of the micropatterned cell monolayers cultured on soft, medium and stiff substrates.

Supplementary Table 2. Parameters adopted in the Monte Carlo simulations.

Supplementary Table 3. Fabrication of PAAm hydrogel substrates with different stiffness.

Supplementary Methods

Supplementary References

Supplementary Figures

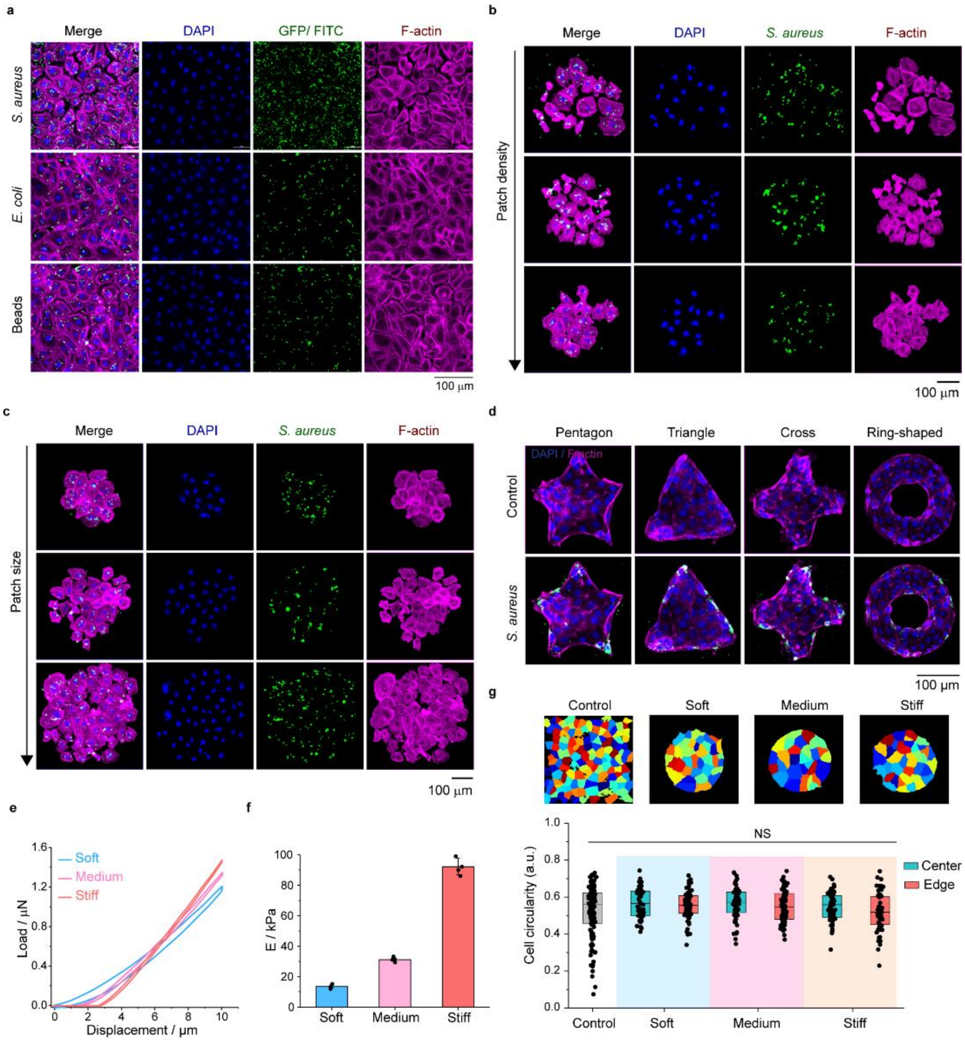

Supplementary Fig. 1 Dependence of spatially heterogeneous bacteria-cell adhesion on geometric constraints. **a** Representative images of IEC-6 cell monolayers without geometric constrains infected by bacteria (*S. aureus and E. coli*) expressing GFP and yellow-green fluorescently labeled latex beads, respectively. For better visualization, nuclei and F-actin cytoskeletons in host cells were also stained simultaneously. Scale bars, 100 μm. **b** Representative images of low-density IEC-6 cell patches infected by *S. aureus*. **c** Representative images of low-density IEC-6 cell patches with different sizes, which were infected with *S. aureus*. **d** Representative images of micropatterned monolayers of IEC-6 cells with different shapes (pentagon, triangle, cross and ring-shaped) before and after infection by *S. aureus* expressing GFP. Scale bars, 100 μm. **e** Representative load-displacement curves of the PAAm substrates with different rigidities (soft, medium and stiff) measured by nanoindentation. **f** The corresponding Young's modulus (*E*) of the PAAm substrates with different rigidities (soft, medium and stiff). **g** Statistical results of cell circularity quantified via the well-developed Cellprofiler software. Top: representative segmentation images of individual cell shapes in a typical cell monolayer without geometric constraints (Control) and micropatterned cell monolayers cultured on the PAAm substrates with different rigidities. Bottom: The corresponding statistical results of circularity of individual cells in the cell monolayer without geometric constraints (Control) and those at the central and edge regions of the micropatterned cell monolayers cultured on the soft, medium and stiff PAAm substrates (n = 3 micropatterns from 3 independent experiments; one-way ANOVA). All representative data were repeated at least three times with similar results. Source data are provided as a Source Data file.

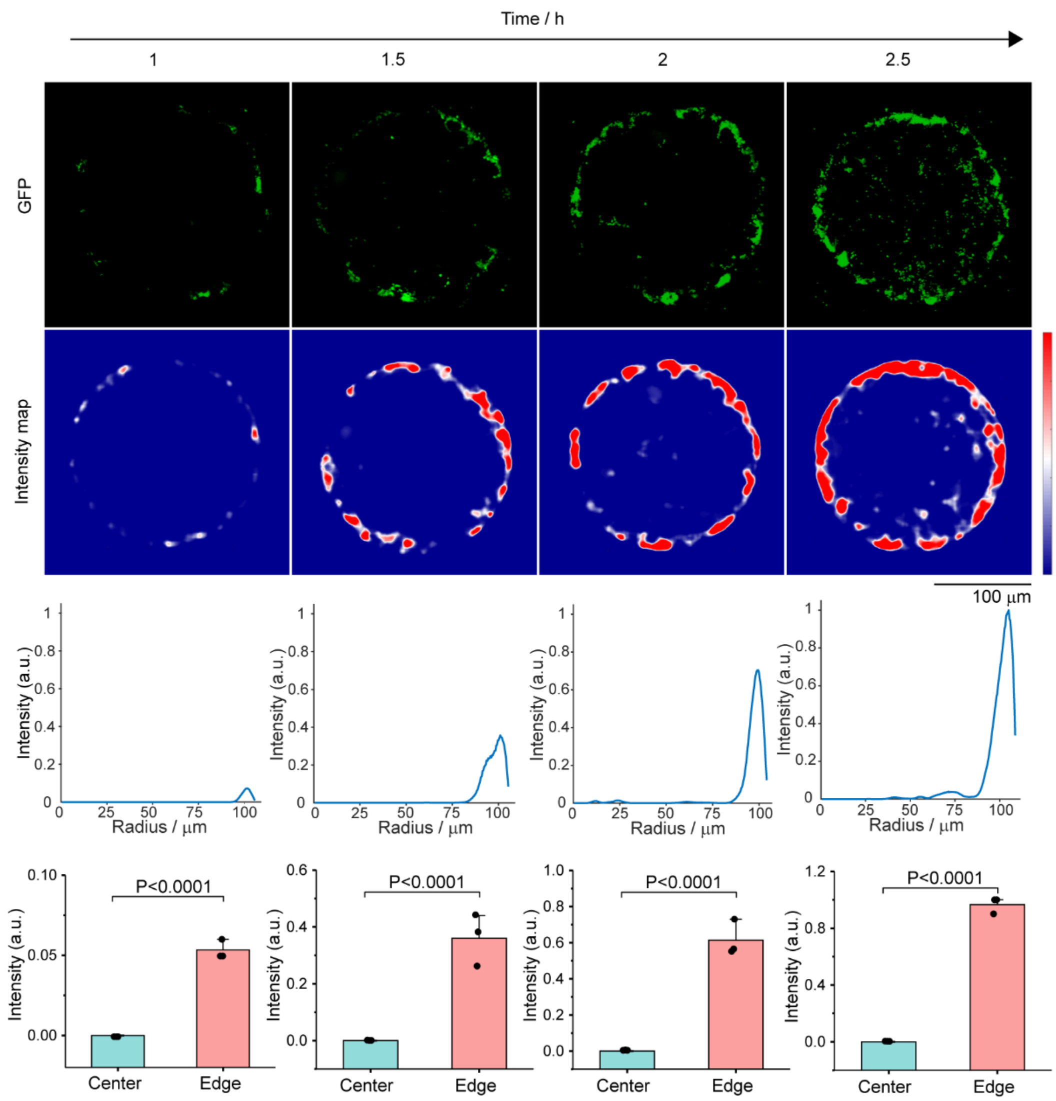

Supplementary Fig. 2 Spatiotemporal distribution of bacterial adhesion to geometrically constrained cell monolayers. Top: spatially heterogeneous adhesion results between *S. aureus* expressing GFP and circular monolayers of IEC-6 cells at 1, 1.5, 2 and 2.5 h of infection. The intensity maps presented spatial distributions of normalized GFP intensities of adherent *S. aureus*. Scale bars, 100 μm. Bottom: average normalized GFP intensities along the radial directions of the micropatterns and statistical comparison of the normalized GFP intensities at the center and the edge of these circular cell monolayers at 1, 1.5, 2 and 2.5 h of infection. All measured values were shown as the mean ± SD (n = 3; two-tailed unpaired t-test). All representative data were repeated at least three times with similar results. Source data are provided as a Source Data file.

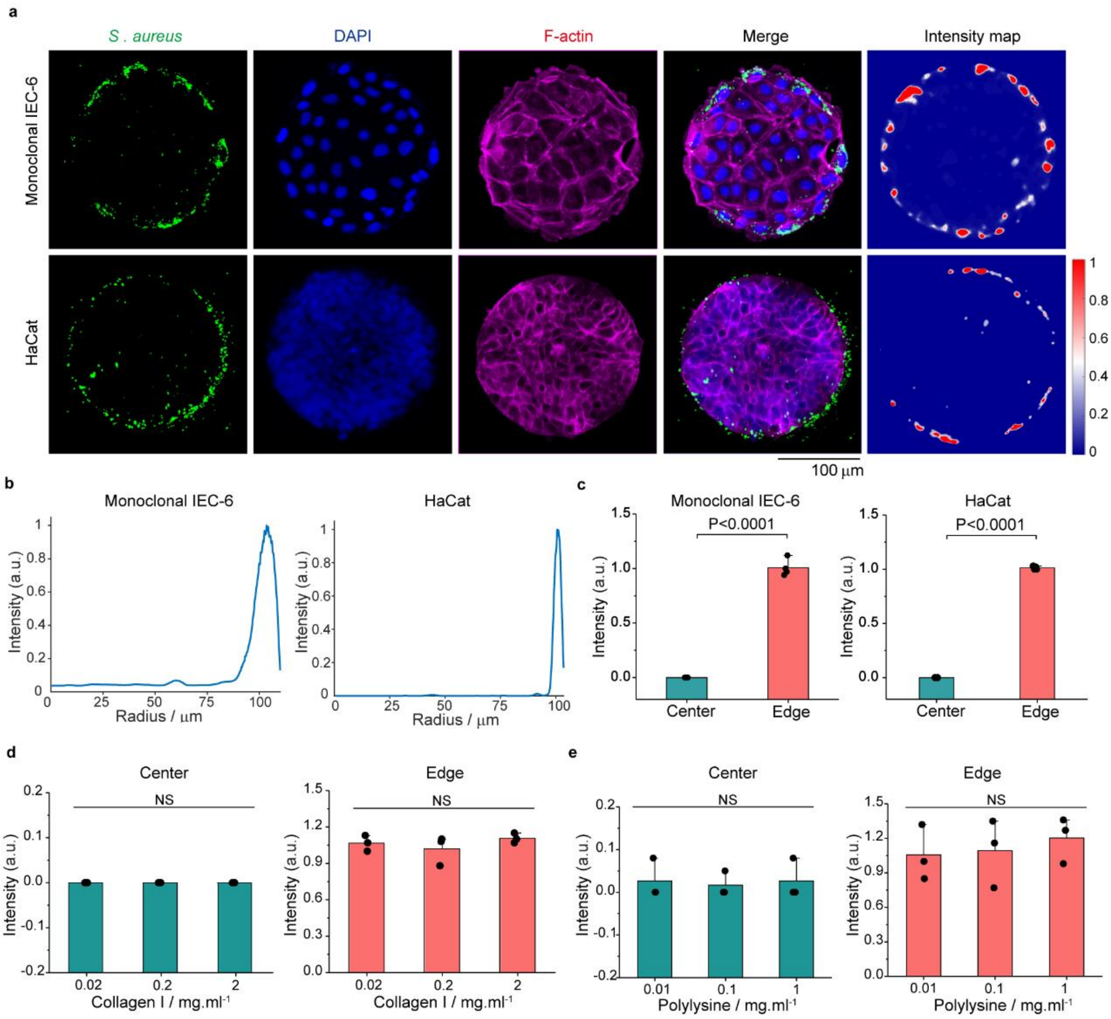

Supplementary Fig. 3 Bacteria-cell heterogeneous adhesion phenomenon independence of cell types and adhesive substance coated on PAAm substrates. a Representative images of circular monolayers of monoclonal IEC-6 cells and HaCat cells infected by *S. aureus* and the corresponding spatial distribution of normalized GFP intensities of adherent *S. aureus*. Note that, in order to exclude the influence of cell phenotype, we allowed a single IEC-6 cell to proliferate within a specific micropatterned region so that it finally formed a micropatterned cell monolayer, which was referred to as monoclonal IEC-6 cell monolayer in our experiments. For better visualization, cell nuclei and F-actin cytoskeletons in the host cell monolayers were labelled with DAPI and Actin-Tracker Red-555 (Thermo Fisher), respectively. Scale bars, 100 μm. **b** Average normalized fluorescence intensities of adherent *S. aureus* along the radial directions of the micropatterns of monoclonal IEC-6 cells and HaCat cells. **c** Statistical comparison of the normalized GFP intensities at the center and the edge of the circular cell monolayers of monoclonal IEC-6 cells and HaCat cells (n = 4; two-tailed unpaired t-test). **d-e** Statistical comparisons of the normalized GFP intensities of adherent *S. aureus* at the center and the edge of the circular cell monolayers of IEC-6 cells on collagen I-coating (**d**) and polylysine-coating PAAm substrates (**e**) (n = 3; one-way ANOVA). All representative data were repeated at least three times with similar results. Source data are provided as a Source Data file.

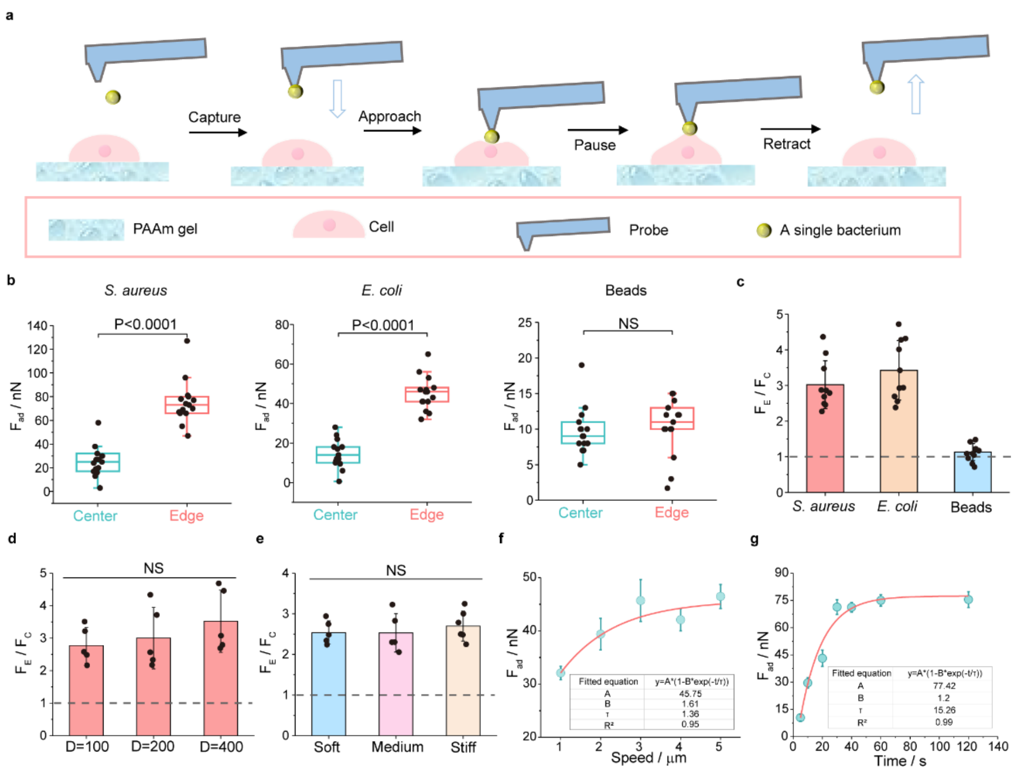

Supplementary Fig. 4 Dependence of adhesion forces between single bacterium and micropatterned host cell monolayers on geometric constrains. a Simplified schematic diagram of measuring adhesion forces between single bacterium and cell monolayers based on FluidFM-based SCFS. First, single bacterium was tightly immobilized to the probe opening by applying negative pressure (-200 mbar). Then, the cantilever with the single bacterium approached a selected adherent cell at a piezo speed of 1 μm/s and paused 30 s to interact with the cell when the force setpoint (100 nN) was reached. Finally, the cantilever was retracted from the cell at a piezo speed of 1 μm/s to complete the force spectrum experiment. **b** Comparison of the measured adhesion forces of *S. aureus*, *E. coli* and latex beads to host cells located at the center and edge of these micropatterned cell monolayers. The underlying PAAm substrate had a stiffness of 93.46 kPa (stiff substrate). Box plots indicate median (middle line), 25th, 75th percentile (box) (two-tailed unpaired *t*-test). **c** Ratio of the adhesion forces of *S. aureus*, *E. coli* and latex beads to host cells located at the edges of the micropatterned cell monolayers to the corresponding ones at the centers (F_E / F_C). **d** Ratio of the adhesion forces of *S. aureus* adhering to the edges of micropatterned cell monolayers with different micropattern diameters (100, 200 and 400 μm) to the corresponding centers (one-way ANOVA). **e** Ratio of the adhesion forces of *S. aureus* adhering to the edges of micropatterned cell monolayers cultured on substrates with different rigidities (soft, medium and stiff) to the corresponding centers. **f** Adhesion forces between *S. aureus* and IEC-6 cell monolayers at different probe speed. **g** Adhesion forces between *S. aureus* and IEC-6 cell monolayers at different probe contact time. All the measured data were shown as mean ± SD ($n \geqslant 3$), unless otherwise stated. Source data are provided as a Source Data file.

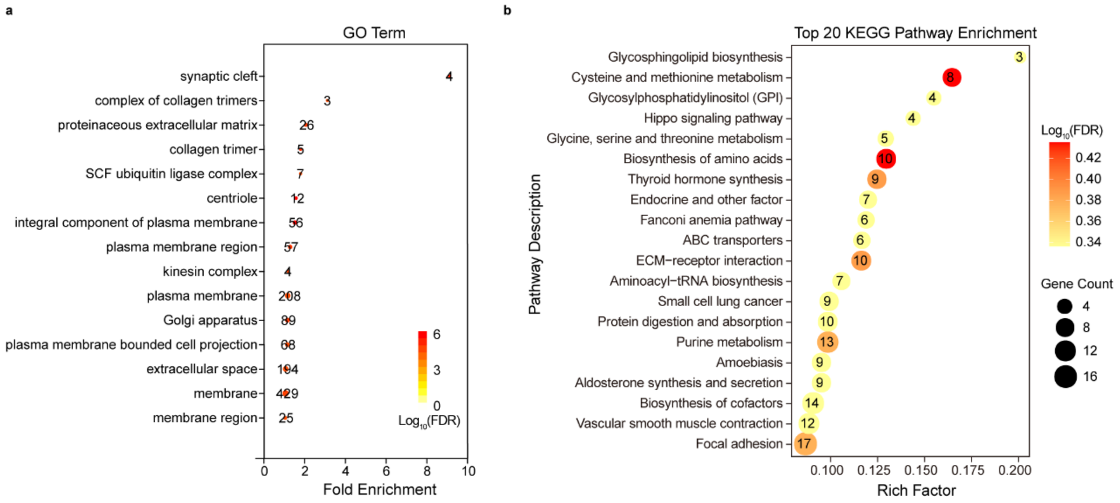

Supplementary Fig. 5 Single-cell RNA sequencing analysis. a Gene ontology (GO) analysis of host cells located at the center and edge of micropatterned monolayers. The bubble chart showed enrichment results of the GO items. The X-axis represented the fold change, the size of circles correlated positively with the number of enriched genes, and the color of circles indicated the significance of enrichment. The fold change denoted the ratio of the FPKM (fragments per kilobase of exon model per million mapped fragments) values of the normalized gene level at the center and edge of the micropatterned cell monolayers. **b** Top 20 Kyoto Encyclopedia of Genes and Genomes (KEGG) pathway analysis of the host cells located at the center and edge of the micropatterned monolayers. The x-axis represented the degree of enrichment. The size of circles correlated positively with the number of enriched genes, and the color of circles indicated the significance of enrichment.

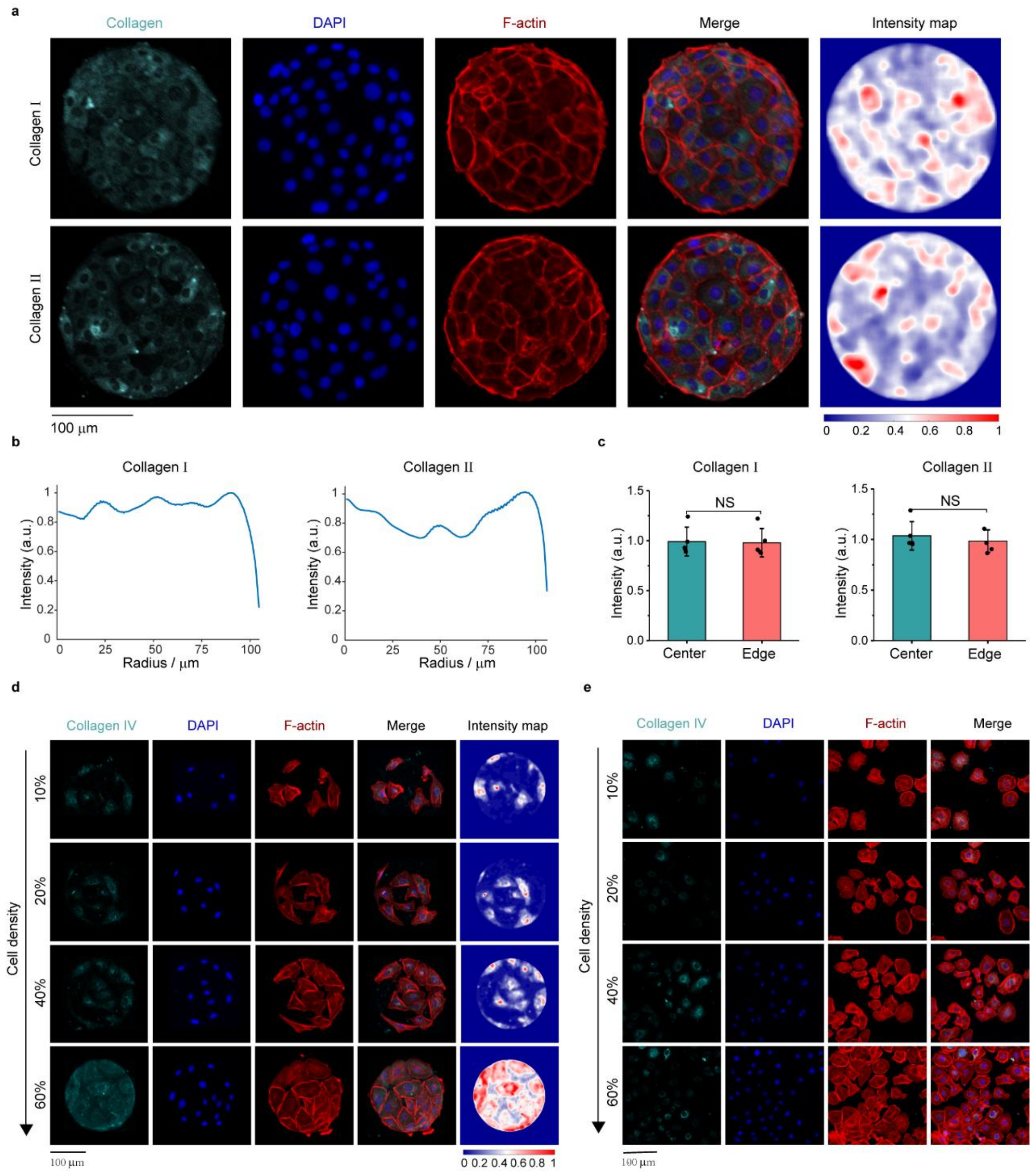

Supplementary Fig. 6 Spatial distribution of collagen I and II on the micropatterned cell monolayers. a Immunofluorescence staining results (cyan) of collagen I and II expressed in the circular cell monolayers cultured on stiff substrates and the corresponding intensity maps. Nuclei and F-actin cytoskeletons in the host cell monolayers were labelled with DAPI and Actin-Tracker Red-555 (Thermo Fisher), respectively. Scale bars, 100 μm. **b** Mean fluorescence intensities of collagen I and II along the radial direction of the circular cell monolayers. **c** Statistical comparisons of average normalized fluorescence intensities of collagen I and II at the center and edge of the circular cell monolayers. **d** Immunofluorescence staining results of collagen IV expression (cyan) in low-density IEC-6 monolayers cultured on the stiff substrates with circular micropatterns. **e** Immunofluorescence staining results of collagen IV expression (cyan) in low-density IEC-6 cells cultured on 12-well plates without geometric constraints. All the measured data were shown as mean ± SD ($n \geqslant 3$), unless otherwise stated. All representative data were repeated at least three times with similar results. Source data are provided as a Source Data file.

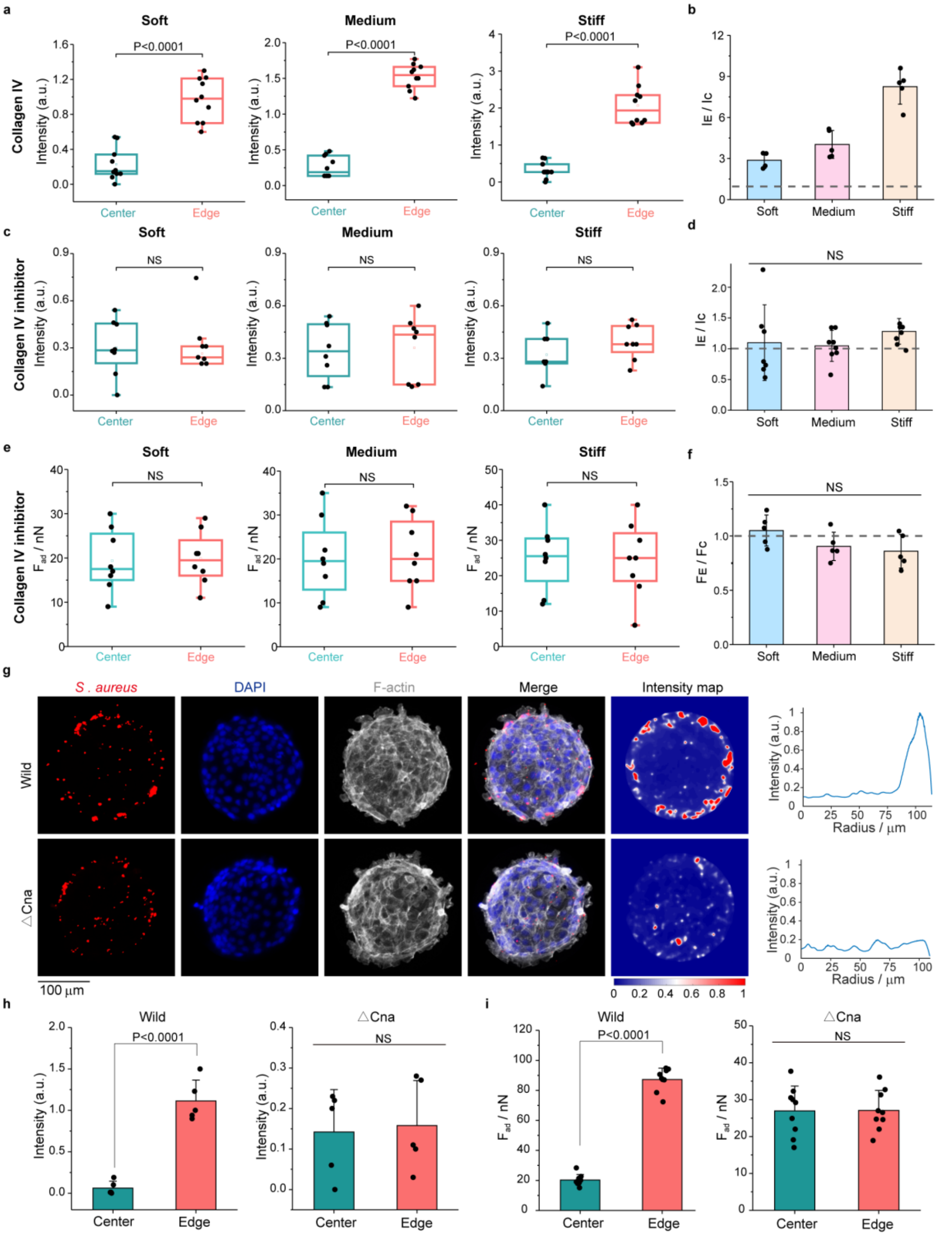

Supplementary Fig. 7 Edge effects of collagen IV on the micropatterned host cell monolayers. a Comparisons of the fluorescence intensities of collagen IV at the center and at the edge of the micropatterned cell monolayers cultured on substrates with different rigidities (soft, medium and stiff). Box plots indicate median (middle line), 25th, 75th percentile (box). **b** Ratio (I_E / I_C) of the fluorescence intensities of collagen IV at the edges (I_E) of micropatterned cell monolayers cultured on substrates with different rigidities (soft, medium and stiff) to the corresponding ones at the centers (I_C). **c** Comparisons of the fluorescence intensities of collagen IV at the center and at the edge of the micropatterned cell monolayers cultured on substrates with different rigidities (soft, medium and stiff) after treatments with the collagen IV inhibitor. **d** Ratio of the fluorescence intensities of collagen IV at the edges of micropatterned cell monolayers cultured on substrates with different rigidities (soft, medium and stiff) after

inhibition of collagen IV to the corresponding ones at the centers (one-way ANOVA). **e** Comparisons of the adhesion forces between *S. aureus* and micropatterned cell monolayers cultured on substrates with different rigidities at the center and at the edge after inhibition of collagen IV. **f** Ratio of adhesion forces of *S. aureus* adhering to the edges of micropatterned cell monolayers cultured on substrates with different rigidities (soft, medium and stiff) after treatments with the collagen IV inhibitor to the corresponding centers (one-way ANOVA). **g** Left: representative images of circular monolayers of IEC-6 cells (200 μm in diameter) infected by *S. aureus* USA300 (wild) and Cna-deficient *S. aureus* USA300 (△Cna), respectively. The bacteria were labelled with pHrodo (ThermoFisher, P36600), whereas nuclei and F-actin cytoskeletons in the host cell monolayers were labelled with DAPI and Phalloidin-iFluor (TM) 647 Conjugate (AAT Bioquest), respectively. Right: normalized fluorescence intensity distribution (heatmap) of bacteria adherent to the micropatterned cell monolayers and the corresponding average fluorescence intensity curves along the radial directions of the micropatterns. **h** Statistical comparisons of average normalized fluorescence intensities of *S. aureus* USA300 (wild) and Cna-deficient *S. aureus* USA300 (△Cna) located at the center and edge regions of the micropatterned cell monolayers ($n = 5$). **i** Statistical comparisons of adhesion forces of *S. aureus* USA300 (wild) and Cna-deficient *S. aureus* USA300 (△Cna) attached to the center and edge regions of the micropatterned cell monolayers ($n = 9$). All the measured data were shown as mean ± SD ($n \geqslant 3$), unless otherwise stated. All representative data were repeated at least three times with similar results. Source data are provided as a Source Data file.

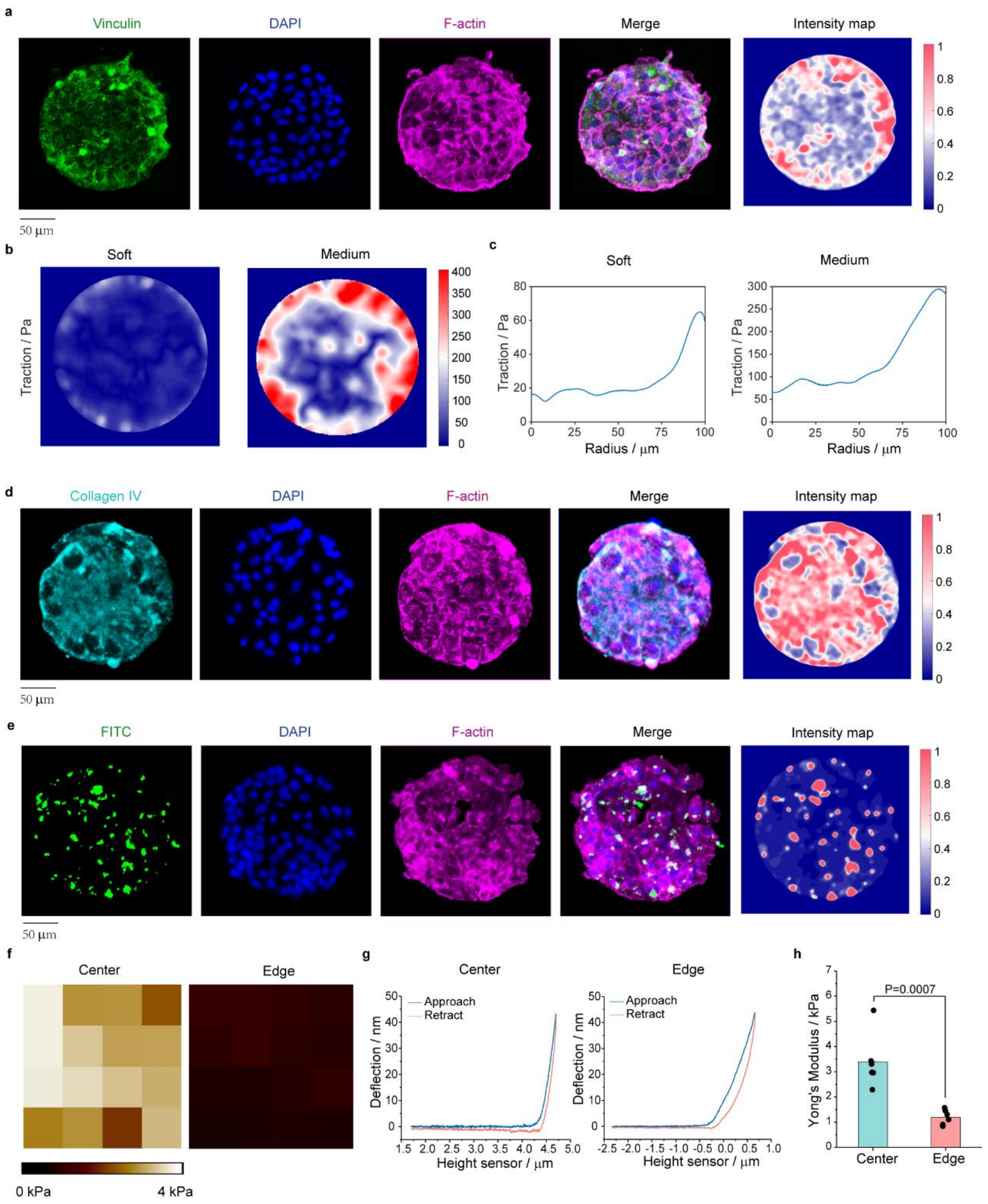

Supplementary Fig. 8 Spatially heterogeneous expression of vinculin and collagen IV in the micropatterned cell monolayers and spatial distribution of cellular traction forces. a Immunofluorescence staining of vinculin in a representative micropatterned cell monolayer and the corresponding fluorescence heatmap displaying its spatial distribution in the cell monolayer. Likewise, nuclei and F-actin cytoskeletons in the cell monolayers were stained with DAPI and Actin-Tracker Red-555 (Thermo Fisher), respectively. **b** Cellular traction forces generated by the micropatterned cell monolayers, which were quantified by the well-established cellular traction force microscopy. The colors showed the absolute magnitude of the traction stresses in Pa. **c** Average magnitudes of celluar traction stresses along the radial direction of the micropatterned cell monolayers grown on PAAm substrates with soft and medium rigidities, respectively. **d** Immunofluorescence staining of collagen IV in a representative micropatterned cell monolayer after cytochalasin D treatment and the corresponding fluorescence heatmap. Also, nuclei and F-actin cytoskeletons in the cell monolayers were labelled with DAPI and Actin-Tracker Red-555 (Thermo Fisher),

respectively. **e** Representative images showing a micropatterned cell monolayer infected by *S. aureus* expressing GFP after cytochalasin D treatment and the corresponding GFP intensity heatmap. For better visualization, nuclei and F-actin cytoskeletons were stained with DAPI and Actin-Tracker Red-555 (Thermo Fisher), respectively. **f-h** Characterization of Young's moduli of the micropatterned cell monolayers based on an atomic force microscope (AFM). **(f)** Spatial distributions of Young's moduli of cells at the center and edge of a representative micropatterned monolayer. **(g)** Typical measurement curves presented by AFM and **(h)** Statistical results of Young's moduli of cells located at center and edge regions of the IEC-6 cell monolayer (n = 6; two-tailed unpaired t-test). All representative data were repeated at least three times with similar results. Source data are provided as a Source Data file.

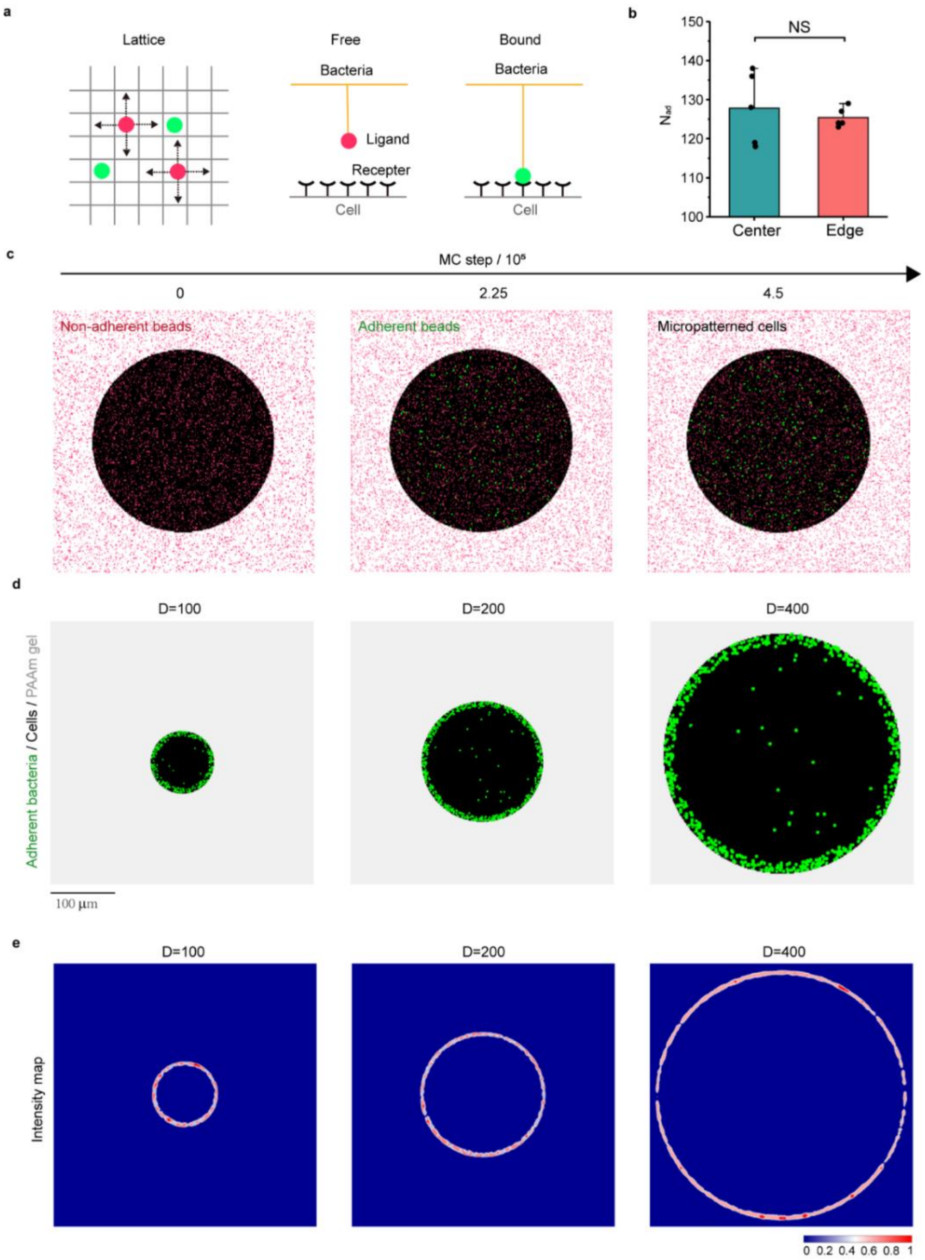

Supplementary Fig. 9 Monte Carlo simulations of bacteria-cell adhesion under geometric constrains. a Schematic diagram of bacteria-cell adhesion in simulation. Non-adherent bacteria were represented by red solid circles and spread freely in the direction of the arrow, while adherent bacteria were represented by solid green circles and bounded to cells to adhere there. **b** Simulation data showing no significant differences in the number of adherent beads onto the center and edge of the circular cell monolayers (n = 5). **c** Typical simulation results characterizing the adhesion between beads and micropatterned cell monolayers at different MC steps. Non-adherent beads were marked as red solid squares, and adherent beads were labelled as green solid squares. Micropatterned cells were denoted with a black background. **d-e** Simulation results presenting spatial distributions (**d**) and corresponding intensity maps (**e**) of adherent bacteria onto the micropatterned cell monolayers with different diameters (100, 200 and 400 μm), where the adherent bacteria were labelled as green solid squares, the micropatterned cell monolayers were marked with a black background, and the underlying PAAm substrates were shown in gray. Scale bars, 100 μm. All representative data were repeated at least three times with similar results. Source data are provided as a Source Data file.

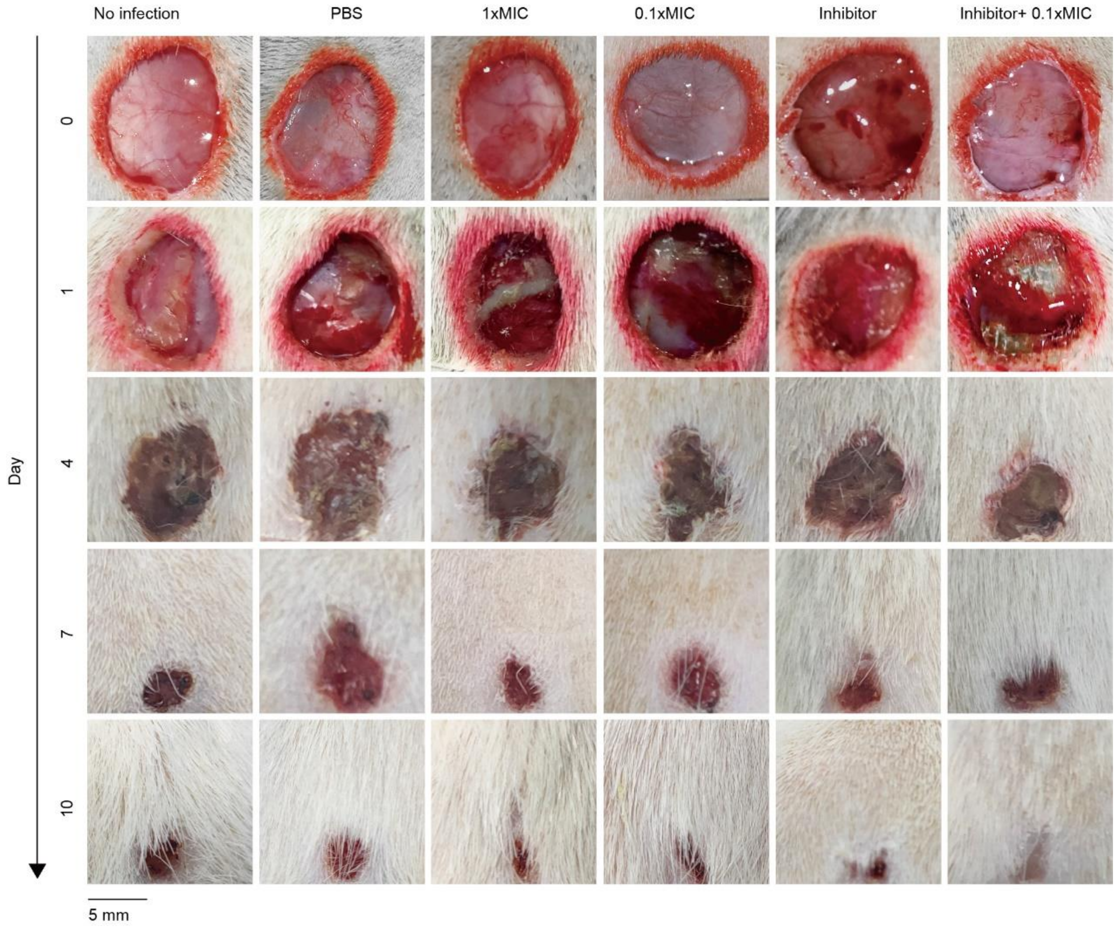

Supplementary Fig. 10 Collagen IV inhibitors enhanced the effect of antibiotics and promoted wound healing. Images of wound closure over time. Scale bars, 5 mm.

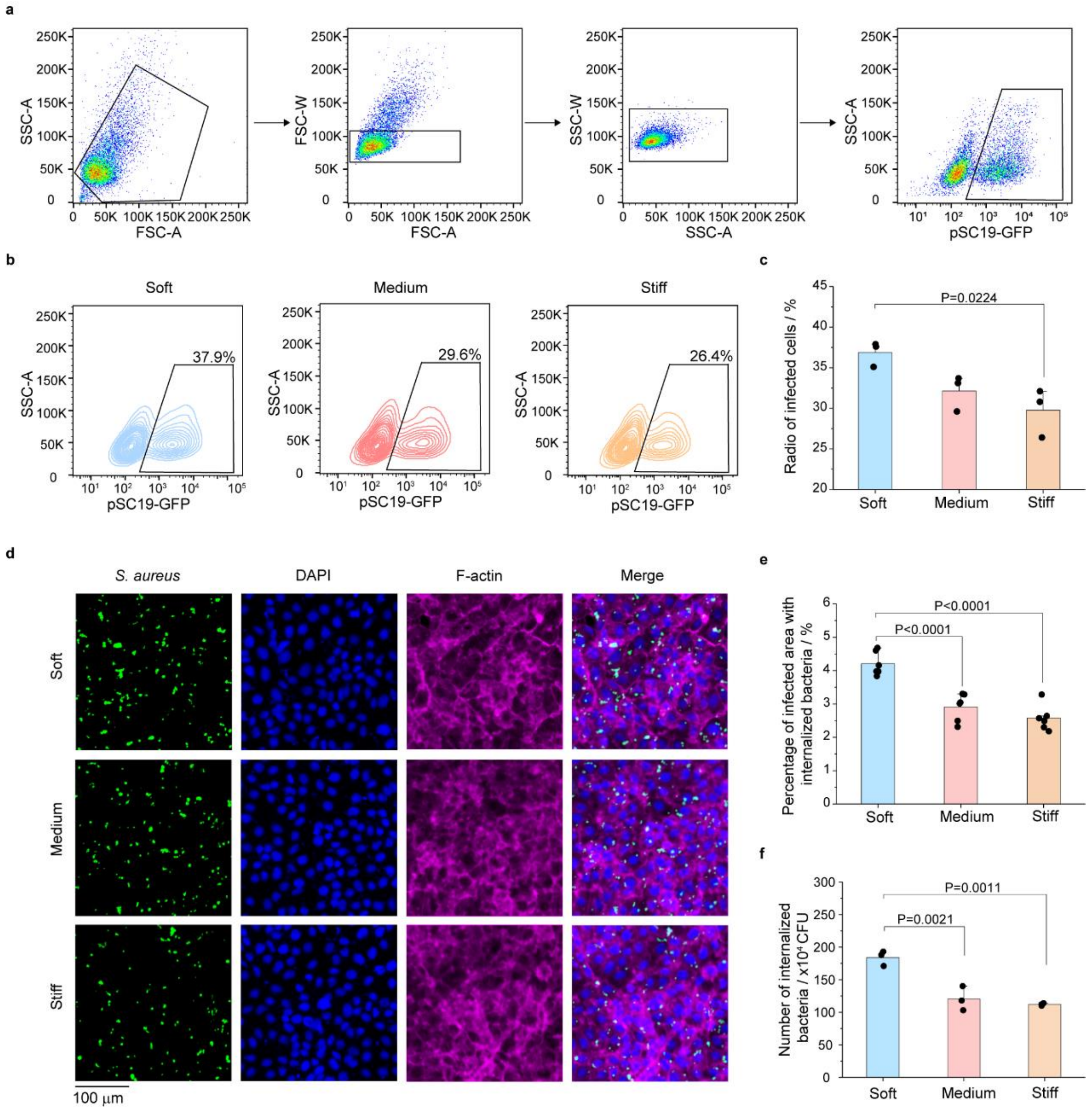

Supplementary Fig. 11 Dependence of invasion of *S. aureus* to IEC-6 cell monolayers on substrate rigidities. **a** Flow cytometry gating strategy for infected cells. The infected and uninfected cells were distinguished according to the GFP fluorescence signals. **b-c** Representative images (**b**) and statistical results (**c**) of cellular infection rate of internalized bacteria on the substrates with different rigidities based on the flow cytometry (one-way ANOVA). **d-e** Representative confocal images (**d**) and statistical results (**e**) of infected area of bacteria internalized to IEC-6 cell monolayers cultured on the substrates with different rigidities (one-way ANOVA). **f** Statistical results of the number of bacteria internalized to IEC-6 cell monolayers cultured on the substrates with different rigidities based on the colony-count technique (one-way ANOVA). All the measured data were shown as mean ± SD ($n \geqslant 3$), unless otherwise stated. Source data are provided as a Source Data file.

Supplementary Tables

Supplementary Table 1. Statistical results of single cell areas at the central and peripheral regions of the micropatterned cell monolayers cultured on soft, medium and stiff substrates.

	**Soft**	**Medium**	**Stiff**
**Central region**	585.35 ± 179.92 μm^2	750.18 ± 301.76 μm^2	587.64 ± 251.25 μm^2
**Peripheral region**	587.13 ± 186.66 μm^2	753.45 ± 299.37 μm^2	652.96 ± 309.18 μm^2

Supplementary Table 2. Parameters adopted in the Monte Carlo simulations.

Abbreviation	Definition	Used value	Source
T	Temperature	310 K	——
R	Radius of single *S. aureus* or beads	0.5 μm	1
ρ	Density of single *S. aureus* or beads	1.1 g/cm^3	2,3
m	Mass of single *S. aureus* or beads	575.5 ng	2
$\mathbf{r}$	Position vector of bacteria or beads	Update over time	——
$\mathbf{v}$	Velocity of bacteria or beads	Update over time	——
η	Liquid viscosity coefficient	0.8 mPa·s	4
ρ_{f}	Liquid density	1.007 g/cm^3	4
$\mathbf{F}_{\mathrm{D}}$	Drag force	Update over time	——
$\mathbf{F}_{\mathrm{B}}$	Brownian random force	Update over time	5
$\mathbf{F}_{\mathrm{G}}$	Gravity of single *S. aureus* or beads	5.64×10^{-15} N	2
$\mathbf{F}_{\mathrm{buo}}$	Buoyancy of single *S. aureus* or beads	5.16×10^{-15} N	4
$k_{\mathrm{a+}}$	Association rate	Update over time	——
$k_{\mathrm{a-}}$	Dissociation rate	Update over time	——
k_{B}	Boltzmann constant	1.38×10^{-23} J/k	——
H	Equivalent height of a collagen molecule	0.21 nm	6
W	Equivalent width of a collagen molecule	8.3 nm	6
E_{c}	Young's modulus of collagen	0.6 GPa	7
E	Young's modulus of the PAAm substrate	10.14-93.46 kPa	Estimation based on experimental data
f	Adhesion force	1-80 nN	Estimation based on experimental data
t	Time	Update over time	——
G_{S}	Size of each lattice	1 μm	——
D	Diameter of micropatterned cell monolayers	100-400 μm	——
A_x	Total number of lattices in x direction	300-600	——
A_y	Total number of lattices in y direction	300-600	——
A_z	Total number of lattices in z direction	50	
N_{a}	Number of adherent bacteria or beads	Update over time	——
N_{non}	Number of non-adherent bacteria or beads	Update over time	——

Supplementary Table 3. Fabrication of PAAm hydrogel substrates with different stiffness.

Elastic Modulus (kPa)	MilliQ water (μl)	Acrylamide (μl of 0.5 mg/mL stock)	Bis-acrylamide (μl of 0.025 mg/mL stock)	APS (μl) of 10% solution	TEMED (μl)
**10.14**	578.50	400	16	5	0.50
**32.29**	636.50	297	61	5	0.50
**93.46**	352.50	282	360	5	0.50

Supplementary Methods

Characterization of PAAm hydrogel stiffness

The elastic modulus of PAAm substrates was quantified by a Piuma nanoindenter equipped with a Chiaro indenter head (Optics11 Life, Netherlands). The adopted nanoindentation probe had a tip radius of 23 μm and its cantilever stiffness was 0.24 N/m in the experiments. At least four nanoindentation tests were performed at different locations on the soft, medium and stiff substrates. According to the obtained load-displacement curves, we employed the following Oliver-Pharr model[8] to estimate the elastic moduli of the PAAm hydrogel samples, *i.e.*,

$$E_{\mathrm{eff}} = \frac{S/2}{\sqrt{R_{\mathrm{i}}}\cdot\sqrt{h_{\max}+h_{\mathrm{r}}}} \tag{1}$$

where E_{eff} was the measured elastic modulus, R_{i} denoted the spherical tip radius, S was the slope at the maximum indentation, $h_{\max}$ represented the maximum indentation depth and h_{r} stood for the final contact depth.

Quantification of cellular traction forces in micropatterned cell monolayers

In general, both singe cells and micropatterned cell monolayers could exert cellular traction forces on extracellular matrices or elastic substrates to which they adhered. The well-developed cellular traction force microscopy (CTFM) provided a powerful tool to recover cellular traction forces[9,10]. In brief, PAAm hydrogel substrates with specific Young's modulus were first fabricated, as described in the Section of Methods. The only difference was that a certain amount of polystyrene fluorescent beads (200 nm in diameter, F-8807, ThermoFisher) needed to be added to the PAAm pre-polymerization solution in advance. Subsequently, a drop of the solution (~30 μl) was added onto a clean coverslip with collagen micropatterns and then covered with another activated coverslip, following a polymerization for 20 min at room temperature. During the time, the fluorescence beads could move to the interface between the PAAm substrate and the clean coverslip under gravity and finally form random fluorescence patterns near the surface of the PAAm substrate. In the experiments, cellular traction forces generated by a specific micropatterned cell monolayer induced the PAAm hydrogel substrate deformation and thus displace the random fluorescent beads near the substrate surface, which could be recorded with a confocal laser scanning microscope (CLSM, Nikon). In this manner, one could obtain the deformed fluorescent image. On the other hand, the originally undeformed fluorescent image could be acquired after the cell monolayer was completely digested with 1 M NaOH. Next, the well-developed digital image correlation (DIC) technique was employed to quantitatively calculate the substrate displacement field by comparing the deformed fluorescent image and the undeformed one[11,12]. Finally, the cellular traction force field was reconstructed from the substrate displacement data by means of the well-established CTFM[13-15].

Quantification of Young's moduli of micropatterned cell monolayers with atomic force microscopy

The Young's moduli of cells in micropatterned cell monolayers were quantified with an atomic force microscope (AFM, BioScope Resolve, Bruker). In the current experiments, the AFM probe had a semi-circular tip whose radius was 970 nm. Prior to the first measurement, the probe was calibrated to determine its actual spring constant (~0.028 N/m). Then, we obtained a series of force-indentation curves at different locations of a specific IEC-6 cell monolayer and estimated their Young's moduli based on the traditional Hertzian model[16], as exhibited in Supplementary Fig. 8f-h.

Bacterial Kinetic Model

To simulate the spatiotemporal dynamics of bacterial interactions with the underlying host cell monolayers, we first introduced a bacterial kinetic model to describe random movement of a single bacterium in a liquid environment. For the sake of simplification, we didn't consider bacterial reproduction and bacteria-bacteria interactions in the model. To facilitate subsequent Monte Carlo (MC) numerical simulations, we divided the two-dimensional square planar area where a host cell monolayer was located into small square grids with side lengths of 1 μm, which was equivalent to the size of a single bacterium such as *S. aureus*, approximately. We assumed that a certain number of bacteria underwent random movements above these delimited square grids under gravity, buoyancy, Stokes drag force, Brownian random force, and eventually came into contact with the underlying host cell monolayer in the liquid environment (Fig. 4c and Supplementary Fig. 9). It was very likely that some of the bacteria approached the underly host cell monolayer and created bacteria-cell adhesion regulated by the receptor-ligand binding. To ensure the efficiency of the MC numerical simulations, we only considered the spatiotemporal dynamics of bacteria within 50 μm above the square region containing a specific host cell monolayer. Simultaneously, we imposed periodic boundary conditions to the cubic simulation regions above the two-dimensional square planar areas, which consisted of 300, 400 and 600 square grids for the simulated micropatterned cell monolayers of 100, 200 and 400 μm in diameter, respectively. If an arbitrary bacterium left the simulation region at one boundary, another new bacterium would immediately enter from the opposite boundary to ensure that the total number of bacteria remained constant during the simulation. For some non-flagellated bacteria that could not move autonomously in a liquid environment[17], such as *S. aureus*, we simplified them to inert colloidal particles[18] in this model. Besides, we further hypothesized that the initial positions and velocities of the simulated bacteria were randomly and uniformly distributed. Based on the Brownian motion theory of suspended particles in a fluid environment[5,19], we might characterize the random motion of a single bacterium under gravity in a liquid environment via a Langevin equation, as presented in Equation (2) in the main text. Namely,

$$m\frac{d^2\mathbf{r}}{dt^2} = \mathbf{F}_\mathrm{D} + \mathbf{F}_\mathrm{B} + \mathbf{F}_\mathrm{G} + \mathbf{F}_\mathrm{buo} \tag{2}$$

in which, m denoted the mass of a single bacterium, $\mathbf{r}$ was its position vector, t was the time, $\mathbf{F}_\mathrm{D}$, $\mathbf{F}_\mathrm{B}$, $\mathbf{F}_\mathrm{G}$ and $\mathbf{F}_\mathrm{buo}$ represented the Stokes drag force, Brownian random force, gravity and buoyancy in the liquid environment, respectively. When the particle speed $\mathbf{v}$ was not large, we had

$$\mathbf{F}_\mathrm{D} = -a\mathbf{v} = -a\frac{d\mathbf{r}}{dt} \tag{3}$$

with

$$a = 6\pi R\eta \tag{4}$$

where a was the Stokes drag coefficient, R denoted the equivalent radius of a single bacterium and η stood for the liquid viscosity coefficient.

In general, the Brownian random force $\mathbf{F}_\mathrm{B}$ was taken as a Gaussian distribution and satisfied the following conditions[5]

$$< \mathbf{F}_\mathrm{B}(t) >= \mathbf{0} \tag{5}$$

$$< \mathbf{F}_\mathrm{B}(t) \cdot \mathbf{F}_\mathrm{B}(t') >= 2ak_\mathrm{B}T\delta（t-t'）\mathbf{I}dt \tag{6}$$

where k_B was the Boltzmann constant, T was the absolute temperature, $\delta(x)$ was the Dirac function and $\mathbf{I}$ was the identity matrix.

The gravity $\mathbf{F}_\mathrm{G}$ acting on a single bacterium could be expressed as

$$\mathbf{F}_\mathrm{G} = mg = \frac{4}{3}\pi R^3 \rho g \tag{7}$$

where R was the radius of the single bacterium, ρ was the density, and g was the acceleration of gravity.

According to the Archimedes' law, the buoyant force $\mathbf{F}_\mathrm{buo}$ exerted on the single bacterium could be quantitatively written as

$$\mathbf{F}_\mathrm{buo} = -\frac{4}{3}\pi R^3 \rho_\mathrm{f} g \tag{8}$$

where ρ_f was the density of the liquid around the bacteria.

Based on the classical Verlet algorithm[20], the position and velocity of a specific bacterium could be calculated in a recursive manner as

$$\mathbf{r}_{(t+\Delta t)} = 2\mathbf{r}_{(t)} - \mathbf{r}_{(t-\Delta t)} + a_t(\Delta t)^2 + \mathbf{O}((\Delta t)^4) \tag{9}$$

$$\mathbf{v}_{(t)} = \frac{1}{2\Delta t}(\mathbf{r}_{(t+\Delta t)} - \mathbf{r}_{(t-\Delta t)}) + \mathbf{O}((\Delta t)^2) \tag{10}$$

Mechanochemical coupling model characterizing bacteria-cell adhesion

To quantitatively describe the kinetics of bacteria-cell adhesion, we put forwards a mechanochemical coupling model to mimic the interaction between collagen IV molecules and collagen-binding proteins (Cna) such as adhesin expressed on *S. aureus* based on the high-affinity “dock, lock and latch (DLL)” mechanism[21-23]. In general, there were three subdomains N1, N2 and N3 in the “A region” in the Cna structure, which was the collagen binding region[24]. Specifically, the N1-N2 subdomain of Cna was a variant of the IgG fold with an open conformation. To form the binding of collagen IV receptors to Cna ligands, the binding groove on the N2 subdomain needed to be docked with the collagen triple helix ligand, then the conformational change locked N1-N2 around the collagen, and finally the C-terminal latch of N2 was inserted into the groove of N1 to latch the ligand[25] (Fig. 4a).

We utilized the classical MC simulations to dissect the spatiotemporal dynamics of random movement of bacteria and possible receptor-ligand binding and dissociation. To this end, we adopted a grid of uniformly sized squares to represent the bacteria-cell adhesion interfaces (Supplementary Fig. 9a). In the simulations, the density of collagen IV proteins on the micropatterned host cell monolayers was assumed to be directly related to the bacteria-cell adhesion energies that were beforehand measured via the FluidFM-based SCFS. The spatiotemporal dynamics of random movement of bacteria and subsequent bacteria-cell interactions was modeled stepwise in the MC simulations until a steady-state equilibrium was achieved. At each time step, spatial positions and velocities of all these bacteria and their states of adhesion to the underlying host cell monolayers were updated accordingly based on Equation (2) and (3) in the main text to determine whether the

ligand-receptor association or dissociation would occur. The spatiotemporal dynamics of interactions between fluorescent beads and the underlying micropatterned cell monolayers was also simulated in a similar manner except that the aforementioned mechanochemical interaction needed to be replaced by potential non-specific interactions, which might be beforehand quantified with the FluidFM-based SCFS.

Quantification of infected cells using flow cytometry

The IEC-6 cell monolayers cultured on the soft, medium and stiff PAAm substrates were infected by *S. aureus* expressing GFP for 2 h. Subsequently, they were washed with PBS to remove unadhered bacteria and then washed twice with 10 mg/ml gentamicin to eliminate uninternalized bacteria without interfering with internalized bacteria[26]. The infected cells were digested with 0.25% trypsin for 3 min, collected with 10% DMEM after centrifugation and resuspended with 3% BSA for the flow cytometry (BD FACSVerse). The cellular infection rate of internalized bacteria was quantified through detecting the GFP fluorescence signals of intracellular bacteria using the flow cytometry.

Quantification of internalized bacteria with colony-count technique

To quantify the internalized bacteria based on the colony-count technique, we also allowed *S. aureus* expressing GFP to infect the IEC-6 cell monolayers cultured on the soft, medium and stiff PAAm substrates for 2 h. Then, the uninternalized bacteria were eliminated with 10 mg/mL gentamicin while the internalized bacteria were released with 0.1% Triton X-100 in experiment. Finally, the internalized bacteria at a certain dilution concentration were smeared on LB agar with glass beads and incubated at 37 ℃ for 18 h to quantify the number of internalized bacteria.

Supplementary References

1. Schwarz-Linek, J. *et al.* Phase separation and rotor self-assembly in active particle suspensions. *Proc. Natl. Acad. Sci. U.S.A.* **109**, 4052-4057 (2012).
2. Feijo Delgado, F. *et al.* Intracellular water exchange for measuring the dry mass, water mass and changes in chemical composition of living cells. *Plos One* **8**, e67590 (2013).
3. Edthofer, A., Novotny, J., Lenshof, A., Laurell, T. & Baasch, T. Acoustofluidic properties of polystyrene microparticles. *Anal. Chem.* **95**, 10346-10352 (2023).
4. Poon, C. Measuring the density and viscosity of culture media for optimized computational fluid dynamics analysis of in vitro devices. *J Mech Behav Biomed Mater* **126**, 105024 (2022).
5. Jung, S. Y. *et al.* Particle deposition on the patterned membrane surface: Simulation and experiments. *Desalination* **370**, 17-24 (2015).
6. Bozec, L. & Horton, M. Topography and mechanical properties of single molecules of type I collagen using atomic force microscopy. *Biophys J* **88**, 4223-4231 (2005).
7. Yang, L., van der Werf, K. O., Dijkstra, P. J., Feijen, J. & Bennink, M. L. Micromechanical analysis of native and cross-linked collagen type I fibrils supports the existence of microfibrils. *J Mech Behav Biomed Mater* **6**, 148-158 (2012).
8. Oliver, W. C. & Pharr, G. M. An improved technique for determining hardness and elastic-modulus using load and displacement sensing indentation experiments. *J. Mater. Res.* **7**, 1564-1583 (1992).
9. Huang, J. Y., Lin, F. & Xiong, C. Y. Mechanical characterization of single cells based on microfluidic techniques. *Trends Analyt Chem* **117**, 47-57 (2019).

10. Huang, J. Y. *et al.* Determination of cellular tractions on elastic substrate based on an integral boussinesq solution. *J Biomech Eng* **131**, 061009 (2009).
11. Yuan, Y., Zhan, Q., Xiong, C. Y. & Huang, J. Y. Digital image correlation based on a fast convolution strategy. *Opt Laser Eng* **97**, 52-61 (2017).
12. Duan, X. C., Yuan, Y., Liu, X. Y., Lin, F. & Huang, J. Y. Coupling self-adaptive meshing-based regularization and global image correlation for spatially heterogeneous deformation characterization. *Exp Mech* **62**, 779-797 (2022).
13. Style, R. W. *et al.* Traction force microscopy in physics and biology. *Soft Matter* **10**, 4047-4055 (2014).
14. Liu, X. *et al.* Extracellular matrix stiffness modulates host-bacteria interactions and antibiotic therapy of bacterial internalization. *Biomaterials* **277**, 121098 (2021).
15. Duan, X. C. & Huang, J. Y. Deep-learning-based 3D cellular force reconstruction directly from volumetric images. *Biophys J* **121**, 2180-2192 (2022).
16. Razvag, Y., Neve-Oz, Y., Sherman, E. & Reches, M. Nanoscale topography-rigidity correlation at the surface of T cells. *Acs Nano* **13**, 346-356 (2019).
17. Samad, T. *et al.* Swimming bacteria promote dispersal of non-motile staphylococcal species. *ISME J* **11**, 1933-1937 (2017).
18. Hermansson, M. The DLVO theory in microbial adhesion. *Colloids Surf. B* **14**, 105-119 (1999).
19. Jung, S. Y. & Ahn, K. H. Transport and deposition of colloidal particles on a patterned membrane surface: Effect of cross-flow velocity and the size ratio of particle to surface pattern. *J Membrane Sci* **572**, 309-319 (2019).
20. Verlet, L. Computer experiments on classical fluids .I. thermodynamical properties of Lennard-Jones molecules. *Phys. Rev.* **159**, 98 (1967).
21. Herman-Bausier, P. *et al.* Mechanical strength and inhibition of the Staphylococcus aureus collagen-binding protein Cna. *Mbio* **7**, e01529-01516 (2016).
22. Couvigny, B. *et al.* Three glycosylated serine-rich repeat proteins play a pivotal role in adhesion and colonization of the pioneer commensal bacterium, Streptococcus salivarius. *Environ. Microbiol.* **19**, 3579-3594 (2017).
23. Ponnuraj, K. *et al.* A "dock, lock, and latch" structural model for a staphylococcal adhesin binding to fibrinogen. *Cell* **115**, 217-228 (2003).
24. Madani, A., Garakani, K. & Mofrad, M. R. K. Molecular mechanics of Staphylococcus aureus adhesin, CNA, and the inhibition of bacterial adhesion by stretching collagen. *Plos One* **12**, e0179601 (2017).
25. Milles, L. F., Schulten, K., Gaub, H. E. & Bernardi, R. C. Molecular mechanism of extreme mechanostability in a pathogen adhesin. *Science* **359**, 1527-1532 (2018).
26. Schirmer, M. *et al.* Linking the human gut microbiome to inflammatory cytokine production capacity. *Cell* **167**, 1125 (2016).
