# Supplementary material for: Geometric constraint-triggered collagen expression mediates bacterial-host adhesion
Source: Nat Commun. 2023 Dec 9;14:8165. doi: 10.1038/s41467-023-43827-6 (PMC10710423; doi:10.1038/s41467-023-43827-6)
Supplement: Supplementary file 3 — Description of Additional Supplementary Files [file 41467_2023_43827_MOESM3_ESM.pdf]

**Description of Additional Supplementary Files**

File Name: Supplementary Movie 1
Description: Quantification of adhesion forces between *S. aureus* and micropatterned IEC-6 cell monolayers based on the FluidFM-based SCFS.

File Name: Supplementary Movie 2
Description: Monte Carlo simulations of spatiotemporal adhesion between *S. aureus* and micropatterned IEC-6 cell monolayers.

File Name: Supplementary Movie 3
Description: Monte Carlo simulations of spatiotemporal adhesion between latex beads and micropatterned IEC-6 cell monolayers.

File Name: Supplementary Movie 4
Description: Monte Carlo simulations of spatiotemporal adhesion between *S. aureus* and micropatterned IEC-6 cell monolayers cultured on substrates with different rigidities.
