# Supplementary material for: Geometric constraint-triggered collagen expression mediates bacterial-host adhesion
Source: Nat Commun. 2023 Dec 9;14:8165. doi: 10.1038/s41467-023-43827-6 (PMC10710423; doi:10.1038/s41467-023-43827-6)
Supplement: Supplementary file 8 — Reporting Summary [file 41467_2023_43827_MOESM8_ESM.pdf]

nature portfolio

Corresponding author(s): Jianyong Huang

Last updated by author(s): Nov 7, 2023

Reporting Summary

Nature Portfolio wishes to improve the reproducibility of the work that we publish. This form provides structure for consistency and transparency in reporting. For further information on Nature Portfolio policies, see our Editorial Policies and the Editorial Policy Checklist.

Statistics

For all statistical analyses, confirm that the following items are present in the figure legend, table legend, main text, or Methods section.

n/a	Confirmed	
☐	☒	The exact sample size (*n*) for each experimental group/condition, given as a discrete number and unit of measurement
☐	☒	A statement on whether measurements were taken from distinct samples or whether the same sample was measured repeatedly
☐	☒	The statistical test(s) used AND whether they are one- or two-sided<br>*Only common tests should be described solely by name; describe more complex techniques in the Methods section.*
☒	☐	A description of all covariates tested
☐	☒	A description of any assumptions or corrections, such as tests of normality and adjustment for multiple comparisons
☐	☒	A full description of the statistical parameters including central tendency (e.g. means) or other basic estimates (e.g. regression coefficient) AND variation (e.g. standard deviation) or associated estimates of uncertainty (e.g. confidence intervals)
☐	☒	For null hypothesis testing, the test statistic (e.g. *F*, *t*, *r*) with confidence intervals, effect sizes, degrees of freedom and *P* value noted<br>*Give P values as exact values whenever suitable.*
☒	☐	For Bayesian analysis, information on the choice of priors and Markov chain Monte Carlo settings
☒	☐	For hierarchical and complex designs, identification of the appropriate level for tests and full reporting of outcomes
☒	☐	Estimates of effect sizes (e.g. Cohen's *d*, Pearson's *r*), indicating how they were calculated

Our web collection on statistics for biologists contains articles on many of the points above.

Software and code

Policy information about availability of computer code

Data collection	Confocal images were acquired using NIS Elements Viewer 5.21. FluidFM BOT software was used for adhesion force measurement. Optics11 Nanoindenter V3.0.28 was used for substrate stiffness measurement. Nanoscpoe 9.4 was used for cell stiffness measurement. Flow cytometric data were collected by BD FACSuite.
Data analysis	Statistical analysis was performed with Origin 2017. Immunofluorescence images are processed and analyzed using ImageJ 1.51k, Matlab 2019a or CellProfiler 4.2.5. Cellular stiffness was based on NanoScope Analysis 1.8. Flow cytometric data were analyzed by FlowJo V10. CaseViewer were used for H&E section analysis.

For manuscripts utilizing custom algorithms or software that are central to the research but not yet described in published literature, software must be made available to editors and reviewers. We strongly encourage code deposition in a community repository (e.g. GitHub). See the Nature Portfolio guidelines for submitting code & software for further information.

Data

Policy information about availability of data

All manuscripts must include a data availability statement. This statement should provide the following information, where applicable:

- Accession codes, unique identifiers, or web links for publicly available datasets
- A description of any restrictions on data availability
- For clinical datasets or third party data, please ensure that the statement adheres to our policy

The single-cell RNA sequencing data used in this study are available in the NCBI SRA database under accession code PRJNA997613 [https://www.ncbi.nlm.nih.gov/bioproject/?term=PRJNA997613]. The remaining data generated in this study are provided in the Supplementary Information or Source Data file. Source data are provided with this paper.

Research involving human participants, their data, or biological material

Policy information about studies with human participants or human data. See also policy information about sex, gender (identity/presentation), and sexual orientation and race, ethnicity and racism.

Reporting on sex and gender	N/A
Reporting on race, ethnicity, or other socially relevant groupings	N/A
Population characteristics	N/A
Recruitment	N/A
Ethics oversight	N/A

Note that full information on the approval of the study protocol must also be provided in the manuscript.

Field-specific reporting

Please select the one below that is the best fit for your research. If you are not sure, read the appropriate sections before making your selection.

☒ Life sciences ☐ Behavioural & social sciences ☐ Ecological, evolutionary & environmental sciences

For a reference copy of the document with all sections, see nature.com/documents/nr-reporting-summary-flat.pdf

Life sciences study design

All studies must disclose on these points even when the disclosure is negative.

Sample size	Throughout the study, the sample sizes are chosen to obtain p-values to determine significant statistical differences and reproducibility of the results.
Data exclusions	No datasets were excluded. We filtered the adhesion forces with a minimum amount to remove spurious points, such as bacteria not adhering to cells or bacteria shedding in the measurement.
Replication	All data are the result of independent biological samples. All the experiments were independently repeated at least three times with similar results.
Randomization	In animal experiments, rats with similar wound sizes were randomized into control and treatment groups. In the in vitro experiments, samples were randomized into experimental and control groups.
Blinding	In in vitro experiments using instrumental readings such as fluidic force microscopy, atomic force microscopy, and flow cytometry, blinding was not implemented due to the inherent objectivity of the testing instruments. However, in cases of possible bias, such as microscopic analysis or animal experiments, the data collectors were blinded to the experimental group.

Reporting for specific materials, systems and methods

We require information from authors about some types of materials, experimental systems and methods used in many studies. Here, indicate whether each material, system or method listed is relevant to your study. If you are not sure if a list item applies to your research, read the appropriate section before selecting a response.

Materials & experimental systems

n/a	Involved in the study
☐	☒ Antibodies
☐	☒ Eukaryotic cell lines
☒	☐ Palaeontology and archaeology
☐	☒ Animals and other organisms
☒	☐ Clinical data
☒	☐ Dual use research of concern
☒	☐ Plants

Methods

n/a	Involved in the study
☒	☐ ChIP-seq
☐	☒ Flow cytometry
☒	☐ MRI-based neuroimaging

Antibodies

Antibodies used

Anti-collagen I (Immunoway, YM3764, 4H10, 1:50), anti-collgen II (Immunoway, YM3749, 1H1, 1:50) , anti-Collagen IV (Novus Biologicals, NB120-6586, 1:50), and anti-vinculin (Bioss, bs-23650R, 1:50) primary antibodies and Alexa Fluor 647-labeled goat anti-mouse secondary antibodies (Beyotime, A0473, 1:200) or Alexa Fluor 488-labeled goat anti-rabbit secondary antibodies (Beyotime, A0423, 1:200) were used.

Validation

The primary antibodies have been validated their respective manufacturers for their respective species, and applications. The validation statement for each primary antibody is available on the manufacturer's website.

Anti-collagen I (4H10) Immunoway, YM3764:
Species: Human, Mouse, Rat; Application: IF, IHC; Manufacturer's website: http://www.immunoway.com/CHome/22/YM3764.

Anti-collgen II (1H1) Immunoway, YM3749:
Species: Human, Mouse, Rat; Application: IF, IHC; Manufacturer's website: http://www.immunoway.com/CHome/22/YM3749.

Anti-collagen IV, Novus Biologicals, NB120-6586:
Species: Human, Mouse, Rat, Bovine, Feline; Application: WB, ELISA, ICC/IF, IHC, IP, IHC, KO, MS; Manufacturer's website: https://www.novusbio.com/products/collagen-iv-antibody_nb120-6586.

Anti-vinculin Bioss, bs-23650R:
Species: Human, Mouse, Rat, Chicken, Dog, Pig, Cow, Horse, Rabbit, Sheep; Application: ELISA, ICC/IF, IHC; Manufacturer's website: http://bioss.com.cn/prolook_03.asp?id=AG09011604422551&pro37=1.

Eukaryotic cell lines

Policy information about cell lines and Sex and Gender in Research

Cell line source(s)

IEC-6 cells (ATCC) were taken from the epithelium of a male rat. HaCat cells (Cell Bank of the Chinese Academy of Sciences) were taken from a male patients aged 62 years.

Authentication

Authentication was assessed by morphology and STR profiling.

Mycoplasma contamination

Cells were regularly tested for mycoplasma and the results were negative.

Commonly misidentified lines
(See ICLAC register)

The HaCat cell line purchased from Beijing zhongkezhijian Biotechnology Co. Ltd. had passed the STR test.

Animals and other research organisms

Policy information about studies involving animals; ARRIVE guidelines recommended for reporting animal research, and Sex and Gender in Research

Laboratory animals

Female SD rats aged 60 days were used.

Wild animals

No wild animals were used.

Reporting on sex

Female rats were used for wound infection experiments in this study, which usually fight little and are easy to raise in groups. Both female and male rats have been reported in wound infection models (Scientific Reports 2022,12,15656; Scientific Reports 2021,11,11678).

Field-collected samples

No field-collected samples.

Ethics oversight

All animal experiments described in this study were complied with the guidelines of Tianjin Medical Experimental Animal Care, and the animal protocols were approved by the Animal Ethics Committee of Yi Shengyuan Genome Technology (Tianjin) Co., Ltd. (protocol number YSY-DWLL-20211031).

Note that full information on the approval of the study protocol must also be provided in the manuscript.

Flow Cytometry

Plots

Confirm that:

☒ The axis labels state the marker and fluorochrome used (e.g. CD4-FITC).

☒ The axis scales are clearly visible. Include numbers along axes only for bottom left plot of group (a 'group' is an analysis of identical markers).

☒ All plots are contour plots with outliers or pseudocolor plots.

☒ A numerical value for number of cells or percentage (with statistics) is provided.

Methodology

Sample preparation	The IEC-6 cell monolayers cultured on the PAAm hydrogels were inected by S. aureus (ATCC29213) expressing GFP for 2 h. Then they were washed with PBS to remove unadhered bacteria and used 10 mg/ml of gentamicin twice to eliminate uninternalised bacteria . The infected cells were digested with 0.25% trypsin for 3 min and collected with 10% DMEM after centrifugation and resuspended with 3% BSA for flow cytometry.
Instrument	BD FACSVerse from Becton Dickinson company.
Software	SD FACSuite software and FlowJo software were used to collect and analyze.
Cell population abundance	N/A
Gating strategy	The gating strategy was detailed in Supplementary Fig. 11a. The infected and uninfected cells were distinguished according to the GFP fluorescence signal.

☒ Tick this box to confirm that a figure exemplifying the gating strategy is provided in the Supplementary Information.
